# Draft genome sequence of yeast *Vanrija* sp. strain TS01, isolated from leukemia patient’s urine

**DOI:** 10.1128/MRA.00152-23

**Published:** 2023-08-23

**Authors:** Lingning Meng, Chang Liu, Liguo Zhu, Xinlei Wang, Chuchu Li

**Affiliations:** 1 Department of Laboratory Medicine, Nanjing Drum Tower Hospital, The Affiliated Hospital of Nanjing University Medical School, Nanjing, Jiangsu, China; 2 Department of Acute Infectious Disease Control and Prevention, Jiangsu Provincial Center for Disease Control and Prevention, Nanjing, Jiangsu, China; University of Maryland School of Medicine, Baltimore, Maryland, USA

**Keywords:** *Vanrija* spp., next-generation sequencing

## Abstract

*Vanrija* sp. strain TS01 was isolated from urine sample from a leukemia patient in Nanjing, China. The closest known relative strain is *Vanrija humicol*a with average nucleotide identity value of 93.1. The draft genome comprises 22.0 Mb in 52 contigs, with G + C content of 62.57%.

## ANNOUNCEMENT

*Vanrija* sp., a species of *basidiomycete* fungi in the family *Trichosporonaceae*, is widely distributed in the environment but seldom reported as a pathogen (1). Here, we report a clinical *Vanrija* sp. strain TS01 which is isolated from a 65-year-old chronic myeloid leukemia patient’s urine.

This organism (TS01) was isolated from a routine urine bacterial culture. After cleaning the patient’s urethral opening, midstream urine was collected using a sterile container and then inoculated onto Columbia blood agar medium (ThermoFisher, MA, USA). After incubation at 35°C for 24 h, white colonies were formed, and microscopic examination revealed that it was a yeast-like fungus. When transferred to Candida chromogenic culture medium (Chromagar, Paris, France) and incubated at 37°C for 24 h, white colonies were formed, making it difficult to identify by colony color. In addition, neither the VITEK 2 COMPACT system nor matrix-assisted laser desorption ionization–time of ﬂight mass spectrometry (Vitek MS with Knowledge Base v3.2; bioMérieux, Inc.) could identify the organism, so sequencing was performed.

After culturing on Columbia blood agar medium for 24 h at 35°C, TS01 cells were lysed using GP1 lysate (Novogene, Beijing, China), and impurities in the lysate were removed by phenol/chloroform/isopentyl alcohol extraction followed by isopropanol precipitation to yield genomic DNA. Sequencing libraries were prepared using the NEBNext Ultra DNA Library Prep Kit for Illumina (NEB, USA), according to the manufacturer’s recommendations. The whole genome of TS01 was sequenced on the Illumina Novaseq 6000 platform using a 2 **×** 150 bp strategy, generating a total of 10,014,137 paired ends. Raw sequencing reads were subjected to quality control and adapter trimming using fastQC v0.11.9 and fastp v0.23.2, respectively (2). Clean reads were then *de novo* assembled using SPAdes v3.15.2 (3), removing contigs smaller than 600 bp to ensure sequencing quality and to meet the requirements for uploading to the NCBI Genome Database, leaving a total of 52 contigs containing 22,668,191 bp. All software was used with default settings unless otherwise specified. The maximum and minimum scaffold lengths were 2,060,691 bp and 604 bp, respectively. The scaffold N50 and N90 values were 895,470 bp and 250,293 bp, respectively. Overall G + C content was determined to be 62.57%.

**FIG 1:**
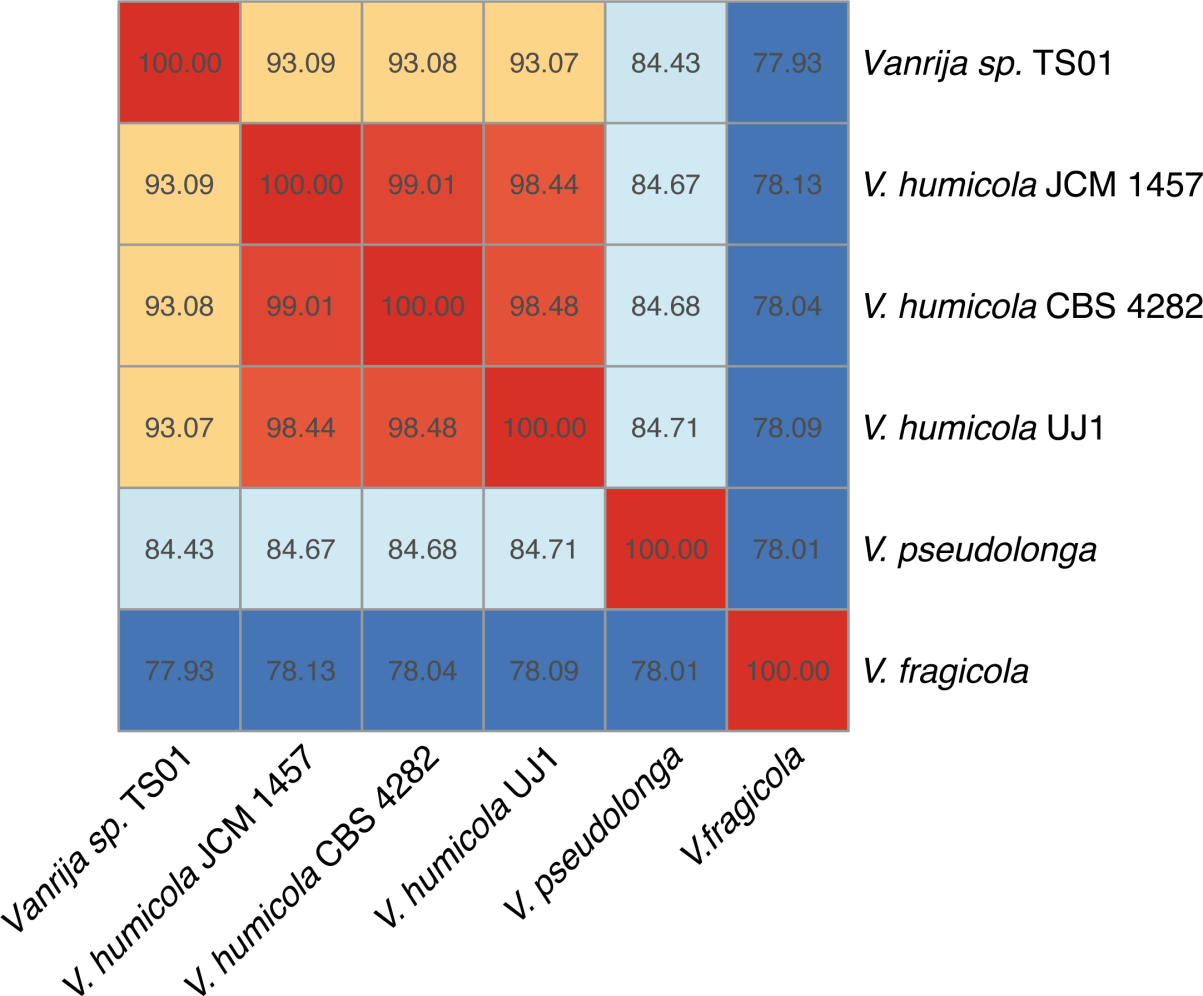
The ANI value matrix of TS01 comparison with other *Vanrija* sp. strains.

Barrnap v0.9 (4) was used to extract the 18s rRNA sequence, NCBI BLASTN results showed that *V. humicola* JCM 1457 had the highest score, indicating that TS01 has a very close relationship with it. Whole-genome average nucleotide identity (ANI) is calculated by FastANI (v1.33) (5), ANI value of TS01 comparison with other *Vanrija* sp. strains like *V. humicola* UJ 1 (GCA_002897395.1) (6), *V. humicola* JCM 1457 (GCA_001600235.1), *V. humicola* CBS 4282 (GCA_008065275.1) (7), *Vanrija pseudolonga* (GCA_020906515.1), and *Vanrija fragicola* (SAMD00084314) is higher than 93% but still below the threshold of 95%. Figure 1 indicates that TS01 belongs to the genus *Vanrija* sp. but is not a member of the species *Vanrija humicola*.

The study has been approved by the Ethics Committee of Nanjing Drum Tower Hospital (approval number: 2023-CR005-01).

## Data Availability

The whole-genome sequencing assembly has been deposited as NCBI BioProject PRJNA898125 with GenBank accession numbers JAPKIE010000001 to JAPKIE010000051 (scaffolds 1 to 51, respectively), and raw reads were uploaded to Sequence Read Archive (SRA) database with SRA accession number SRR23624764. This paper describes the ﬁrst version of the genome.
